# Herpes Simplex Virus Glycoprotein C Regulates Low-pH Entry

**DOI:** 10.1128/mSphere.00826-19

**Published:** 2020-02-05

**Authors:** Tri Komala Sari, Katrina A. Gianopulos, Darin J. Weed, Seth M. Schneider, Suzanne M. Pritchard, Anthony V. Nicola

**Affiliations:** a Department of Veterinary Microbiology and Pathology, College of Veterinary Medicine, Washington State University, Pullman, Washington, USA; b Protein Biotechnology Graduate Training Program, College of Veterinary Medicine, Washington State University, Pullman, Washington, USA; University of Arizona

**Keywords:** herpesviruses, herpes simplex virus, viral entry, viral glycoproteins

## Abstract

Herpes simplex viruses (HSVs) cause significant morbidity and mortality in humans worldwide. Herpesviruses mediate entry by a multicomponent virus-encoded machinery. Herpesviruses enter cells by endosomal low-pH and pH-neutral mechanisms in a cell-specific manner. HSV mediates cell entry via the envelope glycoproteins gB and gD and the heterodimer gH/gL regardless of pH or endocytosis requirements. Specifics concerning HSV envelope proteins that function selectively in a given entry pathway have been elusive. Here, we demonstrate that gC regulates cell entry and infection by a low-pH pathway. Conformational changes in the core herpesviral fusogen gB are critical for membrane fusion. The presence of gC conferred a higher pH threshold for acid-induced antigenic changes in gB. Thus, gC may selectively facilitate low-pH entry by regulating conformational changes in the fusion protein gB. We propose that gC modulates the HSV fusion machinery during entry into pathophysiologically relevant cells, such as human epidermal keratinocytes.

**IMPORTANCE** Herpesviruses are ubiquitous pathogens that cause lifelong latent infections and that are characterized by multiple entry pathways. We propose that herpes simplex virus (HSV) gC plays a selective role in modulating HSV entry, such as entry into epithelial cells, by a low-pH pathway. gC facilitates a conformational change of the main fusogen gB, a class III fusion protein. We propose a model whereby gC functions with gB, gD, and gH/gL to allow low-pH entry. In the absence of gC, HSV entry occurs at a lower pH, coincident with trafficking to a lower pH compartment where gB changes occur at more acidic pHs. This report identifies a new function for gC and provides novel insight into the complex mechanism of HSV entry and fusion.

## Abstract (precis)

Herpesviruses are ubiquitous pathogens that cause lifelong latent infections and that are characterized by multiple entry pathways. We propose that herpes simplex virus (HSV) gC plays a selective role in modulating HSV entry, such as entry into epithelial cells, by a low-pH pathway. gC facilitates a conformational change of the main fusogen gB, a class III fusion protein. We propose a model whereby gC functions with gB, gD, and gH/gL to allow low-pH entry. In the absence of gC, HSV entry occurs at a lower pH, coincident with trafficking to a lower pH compartment where gB changes occur at more acidic pHs. This report identifies a new function for gC and provides novel insight into the complex mechanism of HSV entry and fusion.

## INTRODUCTION

Herpesviruses contain multicomponent fusion complexes and commandeer diverse entry pathways to enter target cells (1–5). Intracellular low pH facilitates entry of several herpesviruses in a cell-specific manner, a concept that was first demonstrated for herpes simplex virus (1, 6–13). HSV, the prototype alphaherpesvirus, utilizes distinct cellular routes to infect its main target cells in the human host (6, 8). HSV enters epithelial cells, the sites of lytic replication by a low-pH mechanism, and neurons, the sites of latent infection, via a pH-neutral one. HSV entry into human keratinocyte cell lines HaCaT and HEKa and into model CHO-HVEM (CHO-herpesvirus entry mediator) cells is inhibited by lysosomotropic agents that elevate the normally low pH of endosomes (Table 1). In contrast, entry into human neural cells IMR-32 and SK-N-SH and into model Vero cells is not blocked by lysosomotropic agents (Table 1).

**TABLE 1:** Cells used in this study and the role of low pH in HSV entry

Cell	Type	Low-pH-dependent entry of HSV^*a*^	Reference
CHO-HVEM	Chinese hamster ovary cell expressing HVEM	Yes	6
HaCaT	Human epidermal keratinocyte	Yes	8
HEKa	Primary adult human epidermal keratinocyte	Yes	8, this study
IMR-32	Human neuroblastoma	No	8
SK-N-SH	Human neuroblastoma	No	8
Vero	African green monkey kidney	No	35

a Inhibited by ammonium chloride, bafilomycin A1, or monensin.

The cellular triggers of herpesvirus entry, including intracompartmental pH, remain incompletely understood. HSV entry requires a host cell receptor that binds to viral glycoprotein D, such as HVEM or nectin-1 (14–16), but additional virus-host interactions are likely critical for entry. HSV particles contain at least 12 different virus-encoded envelope proteins. HSV entry into all cells requires gB, gD, and gH/gL. However, the majority of the remaining viral envelope proteins are not thought to be required for entry via either low-pH or pH-neutral routes (17). Envelope proteins specific for a given HSV entry pathway have not been identified.

Glycoprotein B is conserved among herpesviruses and is a member of the class III fusion protein family. Unlike other class III fusion proteins such as vesicular stomatitis virus (VSV) G and baculovirus gp64, herpesviral gB alone is not sufficient for fusion and requires additional viral proteins, most commonly gH/gL (18). Activation and regulation of the fusion function of gB are incompletely understood. The gH/gL complex is thought to positively regulate gB (19–21). HSV-1 gB undergoes conformational changes during fusion and entry (22, 23). Low pH specifically induces reversible changes in gB domains I and V, which comprise a functional region containing hydrophobic fusion loops (22). Acid-triggered changes in specific gB epitopes correlate with fusion activity as follows: (i) HSV particles entering by endocytosis have reduced reactivity with gB domain I antibody (Ab) H126, and elevation of endosomal pH blocks this change (22); (ii) irreversible acid-triggered changes in the H126 epitope coincide with irreversible acid inactivation of HSV fusion and entry (24); and (iii) a hyperfusogenic form of gB has reduced reactivity with domain I and domain V antibodies, similarly to low-pH-treated gB (25). Thus, the acidic milieu of endosomes may serve as a host cell trigger of gB function.

HSV-1 gC, a 511-amino-acid, type I integral membrane glycoprotein, mediates HSV-1 attachment to host cell surface glycosaminoglycans. This interaction is not essential for HSV entry (26–28). Here, we report that gC regulates low-pH viral entry independently of its known role in cell attachment. We demonstrate that gC facilitates low-pH-induced antigenic changes in gB and that gC enhances the ability of HSV to enter and infect cells by a low-pH pathway. The results are consistent with the following model: in the absence of gC, HSV entry occurs at a lower pH, coincidently with trafficking to a lower pH compartment where gB changes occur at more-acidic pHs. We propose that gC modulates HSV entry mediated by gB, gD, and gH/gL into physiologically relevant cell types, such as human keratinocytes.

## RESULTS

### HSV-1 gC facilitates entry and infectivity of cells that support a low-pH entry mechanism.

HSV envelope glycoproteins gB, gD, and gH/gL are required for entry into all cell types regardless of whether intracellular pH is important for entry in a particular cell type (7, 29–32). A survey of seven additional viral envelope proteins indicated that HSV gE, gG, gI, gJ, gM, UL45, and Us9 are dispensable for entry mediated by either low-pH or pH-neutral pathways (17). In this study, we probed the role of gC in low-pH entry by employing an HSV-1 KOS strain with the gC gene deleted (HSV-1 ΔgC2-3 or ΔgC) and a repaired version of this virus containing the wild-type gC gene (HSV-1 gC2-3R or gCR) (33).

HSV-1 gC is widely recognized to initiate the viral entry process by attaching to host cell surface glycosaminoglycans, principally heparan sulfate proteoglycans (28, 34). When gC-negative HSV-1 is added to cells, there is a delay in entry from 20 to 60 min postinfection (p.i.) relative to wild-type HSV-1 (26). However, by 90 min p.i., the levels of penetration of wild-type and gC-null viruses are indistinguishable. HSV-1 lacking gC has a 1 log defect in infectivity (26). Thus, while gC is dispensable in cell culture, it remains important for the viral replicative cycle.

**FIG 1:**
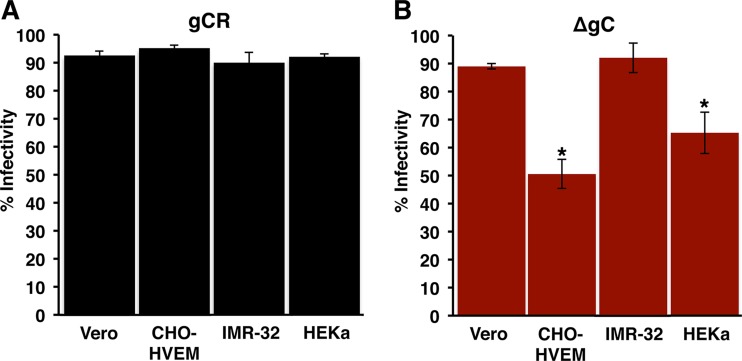
HSV-1 lacking gC exhibits reduced entry and infectivity in a subset of cell types. Equivalent inocula of HSV-1 gCR (A) or ΔgC (B) were bound to Vero, CHO-HVEM, IMR-32, or HEKa cells for 1 h at 4^0^C. Following a shift to 37°C for 6 h, infected cells (MOI of ∼0.9) were quantitated by immunofluorescence. Infectivity is reported as percent HSV antigen-positive cells of ∼500 total cells. Data represent means of results from three independent experiments each performed in triplicate, with standard deviations. *, *P < *0.05 (Student's *t* test).

The contribution of gC to entry of HSV-1 by a low-pH pathway was evaluated. HSV entry into CHO receptor cells and human keratinocytes proceeds via a low-pH endocytic pathway and is well characterized (Table 1) (6, 8). The efficiency of HSV-1 ΔgC infection of CHO-HVEM cells and primary human keratinocytes (HEKa) was compared to the levels seen with Vero and IMR-32 cells, which support pH-neutral entry via penetration at the plasma membrane (8, 35, 36). To control for attachment, virus was first added to cells at 4°C for 1 h. Following a shift to 37°C for 6 h, the percentage of viral antigen-positive cells was determined. The levels of efficiency of HSV-1 gCR entry into each of the four cell types were similar under the conditions tested (Fig. 1A). In contrast, entry of HSV-1 ΔgC into CHO-HVEM and HEKa cells was ∼50% and 35% less efficient than entry into Vero and IMR-32 cells, respectively (Fig. 1B). This suggests that gC contributes to entry of HSV into CHO-HVEM cells and primary human keratinocytes.

### gC contributes to HSV plating efficiency on cells that support a low-pH entry pathway.

**FIG 2:**
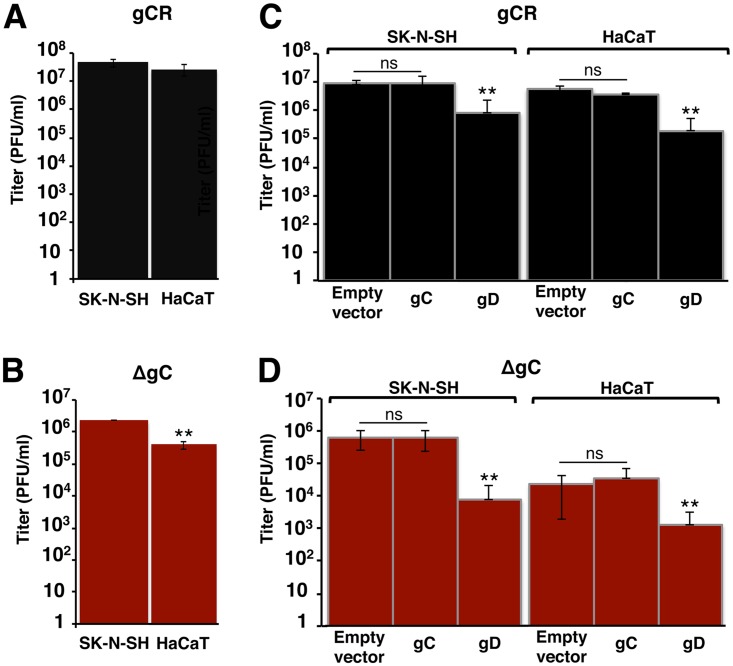
Efficiency of gC-negative HSV-1 infection of human cells that support pH-neutral or low-pH entry. (A and B) HSV-1 gCR (A) and ΔgC (B) titers were determined on SK-N-SH or HaCaT cells by plaque assay. (C and D) Attempt to restore infectivity of HSV-1 lacking gC by providing gC in the cell. Equivalent inocula of HSV-1 gCR (C) or ΔgC (D) were added to SK-N-SH or HaCaT cells transfected with empty vector or gC or gD plasmids at 37^0^C. At 18 to 24 h p.i., titers were determined by plaque assay. Data represent means of results from three independent experiments performed in at least triplicate with standard deviations. **, *P < *0.01; ns, not significant [Student's *t* test]).

To confirm and extend this observation using an alternative approach, the plating efficiency of HSV-1 ΔgC on different human cell lines was tested. The neuroblastoma SK-N-SH line supports pH-neutral entry of HSV, and HaCaT epidermal keratinocytes (EK) support low-pH entry (Table 1) (8). Titers of identical preparations of HSV-1 ΔgC and gCR were determined. The levels of gCR plating efficiency on SK-N-SH and HaCaT cells were similar (Fig. 2A). HSV-1 ΔgC showed ∼1 log lower plating efficiency on HaCaT cells than on SK-N-SH cells (*P* < 0.01) (Fig. 2B). This suggests that gC is specifically important for HSV infectivity of HaCaT cells.

To investigate a potential mechanism of the involvement of gC in low-pH entry, we determined whether ectopic expression of gC restored the infectivity defect of HSV-1 ΔgC in HaCaT cells. SK-N-SH or HaCaT cells were transfected with gC or gD plasmids, and then the plating efficiency of HSV-1 gCR or ΔgC was determined. Cellular expression of gC had no effect on the infectivity of HSV-1 gCR or ΔgC (Fig. 2C and D). Cell-expressed gD reduces HSV entry and infectivity by competing with virion gD for receptor binding (37). As expected, ectopic expression of gD in either of the cell types reduced infectivity of both HSV-1 gCR and HSV-1 ΔgC (Fig. 2C and D).

### gC has no detectable effect on the protein composition of HSV particles.

To address the possibility that deletion of gC might affect the incorporation of gB or other viral proteins into the HSV particle, the protein composition of HSV-1 ΔgC was compared to that of gCR. The absence of gC from virions did not measurably alter the protein composition of particles as measured by sodium dodecyl sulfate-polyacrylamide gel electrophoresis (SDS-PAGE) and protein staining (see Fig. S1A in the supplemental material). Envelope proteins gB, gD, gE, and gH were detected at equivalent levels in the two viruses by Western blotting (Fig. S1B) (38). As expected, gC was not detected in HSV-1 ΔgC virions. The rescuant gCR contains an amount of gC equivalent to that measured for the wild-type KOS parent (33) (data not shown). These results suggest that the defective phenotypes of HSV-1 ΔgC are not explained by indirect effects of the incorporation of gB, gD, or gH into viral particles and are consistent with gC playing a specific role in low-pH entry and infectivity of HSV.

10.1128/mSphere.00826-19.1 FIG S1 Protein composition of gC-negative HSV-1 ΔgC. Equivalent levels of VP5 units of HSV-1 gCR or ΔgC in Laemmli buffer were separated by SDS-PAGE followed by (A) protein staining with Coomassie blue or (B) Western blotting for the indicated viral proteins. Panel B is adapted from a figure in reference 38. Molecular weight standards are indicated in kilodaltons to the left. Download FIG S1, TIF file, 0.6 MB. Copyright © 2020 Komala Sari et al. 2020 Komala Sari et al. This content is distributed under the terms of the Creative Commons Attribution 4.0 International license.

### Effect of ammonium chloride on low-pH entry of ΔgC HSV.

**FIG 3:**
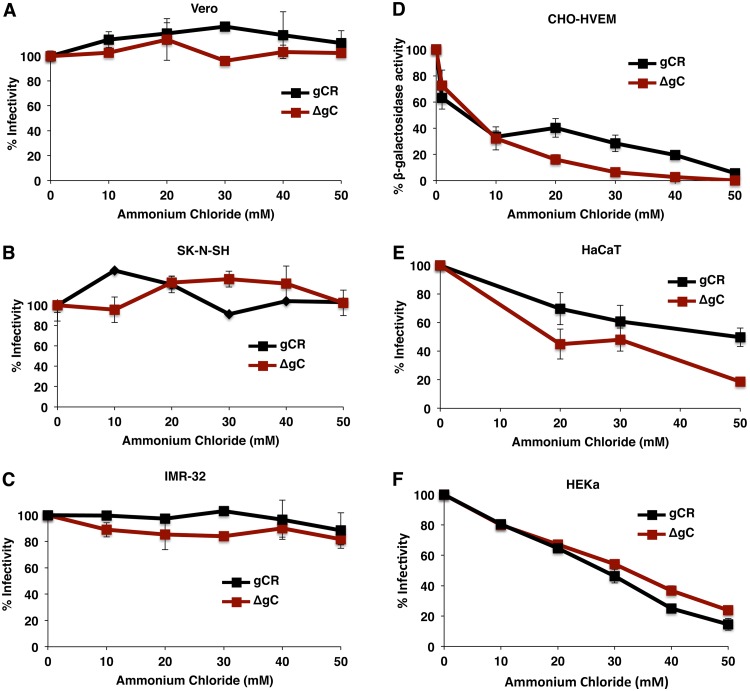
HSV-1 ΔgC enters cells via a low-pH-dependent pathway. Vero (A), SK-N-SH (B), IMR-32 (C), HaCaT (E), or HEKa (F) cells were treated with ammonium chloride for 1 h at 37^0^C. Cells were infected with 100 PFU of HSV-1 ΔgC or gCR for 6 h in the continued presence of drug. Normal medium was added, and at 22 h p.i., infectivity was determined by plaque assay. The infectivity of no-drug samples was set to 100%. (D) CHO-HVEM cells were treated with ammonium chloride for 20 min at 37^0^C. HSV-1 gCR or HSV-1 ΔgC was added to cells (MOI of 5) at 37°C in the continued presence of agent. At 6 h p.i., entry was measured as a percentage of beta-galactosidase activity obtained in the absence of ammonium chloride. Data represent means and standard deviations of results from quadruplicate samples and are representative of at least two independent experiments.

The members of the quartet of gB, gD, and gH/gL are essential for pH-neutral and low-pH entry. gC is dispensable for pH-neutral entry (39). To determine whether gC is essential for low-pH-dependent entry of HSV, we tested the effect of ammonium chloride treatment of CHO-HVEM, HaCaT, and HEKa cells on HSV-1 ΔgC entry using a reporter assay for entry. Ammonium chloride blocks wild-type HSV entry into these cells (Table 1). Ammonium chloride inhibited HSV-1 ΔgC entry into CHO-HVEM, HaCaT, and HEKa cells in a concentration-dependent manner (Fig. 3D to F). HSV-1 gCR was similarly inhibited. Together, the results suggest that gC contributes to initial infection of cells that support a low-pH entry pathway (Fig. 1 and 2) but is not by itself a viral determinant of pathway selection (Fig. 3). Ammonium chloride had little to no inhibitory effect on HSV-1 gCR entry into Vero, SK-N-SH, or IMR-32 cells (Fig. 3A to C), which is consistent with pH-neutral entry of wild-type HSV in these cells (Table 1). Entry of HSV-1 ΔgC into Vero, SK-N-SH, or IMR-32 cells was similarly unaffected by ammonium chloride (Fig. 3A to C), consistent with the notion that gC is dispensable for pH-neutral entry of HSV (39).

### gC does not contribute to viral attachment under the conditions tested.

**FIG 4:**
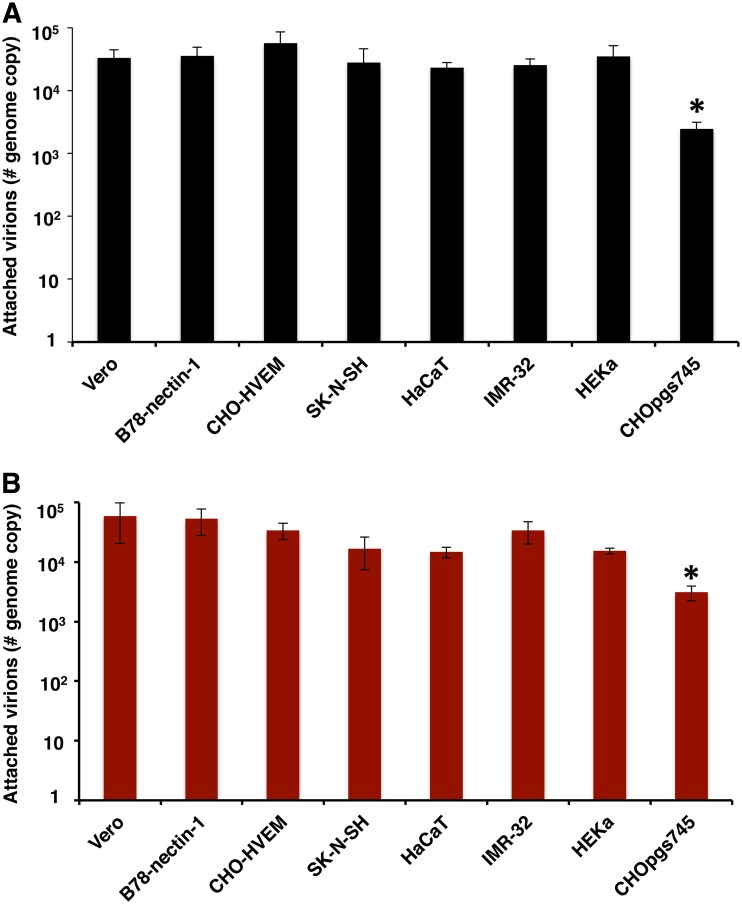
HSV-1 ΔgC attachment to cells. (A and B) Approximately 10^6^ genome copies of extracellular HSV-1 gCR (A) or ΔgC (B) were added to the indicated prechilled cell monolayers at 4°C for 1 h on ice. Following two PBS washes, cells were trypsinized, and cell-associated HSV-1 was quantitated by qPCR. CHOpgs745 cells lack heparan sulfate receptors for HSV attachment and served as controls. Data represent means of results from three independent experiments performed in quadruplicate with standard errors. *, *P < *0.01 (one-way analysis of variance [ANOVA]).

The experiments described in this report were designed to exclude or limit the contribution of viral attachment to the entry and infectivity results. We assessed the role of gC in HSV-1 attachment to each of the cell types under the experimental conditions used in this study. HSV-1 ΔgC or gCR was added to Vero, CHO-HVEM, SK-N-SH, HaCaT, IMR-32, or HEKa cells on ice for 1 h at 4°C. Cell-attached HSV-1 levels were analyzed by quantitative PCR (qPCR). HSV-1 ΔgC attached to all cells in a manner similar to that seen with HSV-1 gCR (Fig. 4). CHOpgs745 cells lack a gene required for heparan sulfate biosynthesis. Both viruses exhibited defective attachment to control CHOpgs745 cells (Fig. 4). These results suggest that the altered entry and infectivity phenotypes of HSV-1 ΔgC seen under the conditions tested here cannot be explained by a defect in HSV-1 attachment.

### gC drives the kinetics of viral penetration from acidic vesicles following intracellular transport of endocytosed HSV.

**FIG 5:**
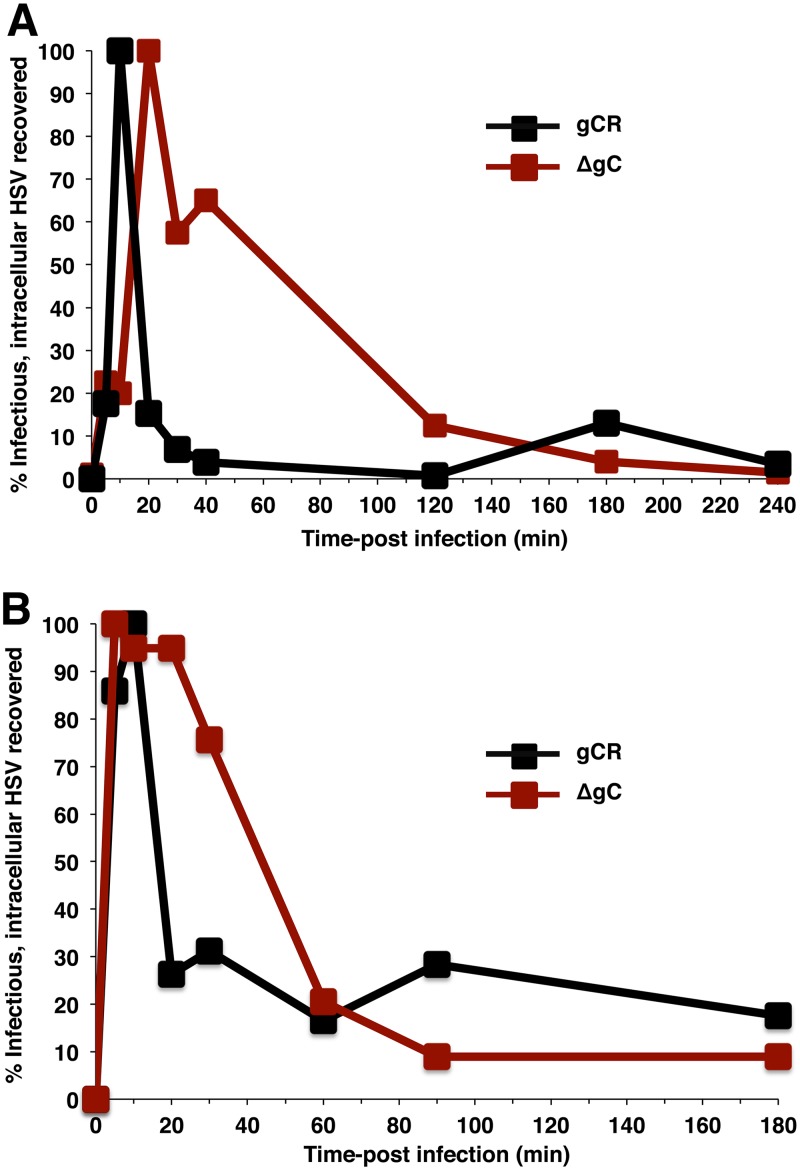
gC contributes to rapid HSV penetration following endocytosis. HSV-1 ΔgC or gCR was bound to CHO-HVEM (A) or HEKa (B) cells for 1 h at 4°C (MOI of 8). Following a shift to 37^0^C, extracellular virions were subjected to citrate inactivation at the indicated times p.i. Titers of freeze-thaw cell lysates were determined on Vero cells as an indication of infectious, enveloped, intracellular particles. This allows monitoring of viral trafficking and penetration over time. Peak recovery titers were set to 100%. Data are representative of results from at least two independent experiments.

To probe further the role of gC in low-pH entry, we monitored the kinetics of intracellular transport of HSV-1 (7). Virus was attached to cells at 4°C for 1 h. Cultures were then shifted to 37°C, and at different times postinfection (p.i.), the remaining extracellular virions were inactivated by citrate treatment, and cells were lysed by two freeze-thaw cycles. Titers of lysates were determined on Vero cells. The detection of infectious HSV in cell lysates reflects the presence of enveloped HSV within cellular endocytic compartments. As expected, HSV uptake into vesicles was rapid. At 10 min p.i. in CHO-HVEM cells, there was a peak of intracellular, infectious gCR virus (Fig. 5A). Following endocytic uptake, HSV fuses rapidly with the endosomal membrane and releases its capsid into the cytosol (7). This was reflected in the sharp decrease in the level of infectious enveloped gCR recovered by 20 min p.i. (Fig. 5A). Interestingly, ∼50% of infectious, intracellular HSV-1 ΔgC was recovered as late as 40 min p.i., suggesting a delay in HSV fusion with endocytic compartments in the absence of gC (Fig. 5A). Analysis of HSV-1 gCR and ΔgC trafficking in HEKa cells yielded results similar to those seen with CHO-HVEM cells (Fig. 5B). For HSV-1 ΔgC entry into the primary human keratinocytes, there was an ∼40-min lag in the intracellular transport and exit of HSV relative to HSV-1 gCR (Fig. 5B). The results presented in Fig. 5 suggest an important postattachment role for gC in the first ∼20 min of wild-type infection. The absence of gC appears to have been overcome by ∼60 to 120 min p.i., perhaps reflecting the reason that gC is not absolutely essential for low-pH entry when longer-term assays are employed. An alternative possibility is that gC-null viruses lose recoverable infectivity because they are degraded whereas wild-type and rescue HSVs lose recoverability because they complete the fusion process. Together, the results suggest that gC mediates rapid intracellular transport of enveloped HSV or may aid in rapid exit of HSV from acidic intracellular vesicles.

### gC positively regulates low-pH-induced antigenic changes in gB.

**FIG 6:**
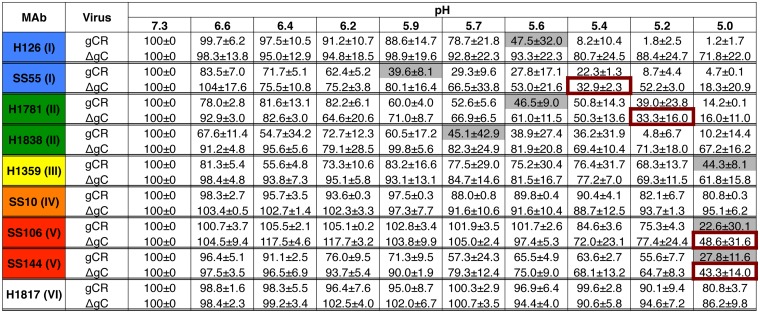
HSV-1 gC influences the pH of conformational changes in the fusion protein gB. Extracellular preparations of HSV-1 gCR or ΔgC (∼10^7^ genome copies) were treated at pH levels ranging from 7.3 to 5.0 and blotted directly to nitrocellulose. Blots were probed with gB MAbs at neutral pH. Antibody reactivity was quantitated with ImageJ. The reactivity of pH 7.3 samples was set to 100%. Data represent means and standard deviations of results from at least two independent experiments. The pH treatment that reduced reactivity by >50% is indicated with gray shading (gCR) or red boxes (ΔgC). After each MAb designation, the gB domain (I to VI) containing the MAb epitope is indicated in parentheses (see Fig. S4 in the supplemental material).

To further delineate the mechanism underlying the role of gC in low-pH entry, the effect of gC on low-pH-triggered antigenic changes in gB was assessed. The prefusion conformation of gB in the virion envelope undergoes low-pH-triggered changes in gB domains I and V (22). These changes are at least partially reversible (22, 40, 41) and are thought to be important for membrane fusion (22, 24, 25). Domain I of gB contains internal hydrophobic fusion loops that are critical for membrane fusion (42, 43). HSV-1 ΔgC or gCR virions were treated with a range of mildly acidic pHs (5.0 to 7.3) and blotted immediately to nitrocellulose membrane. Blots were probed at neutral pH with nine monoclonal antibodies (MAbs) to distinct epitopes in gB, and antibody reactivity was detected and quantitated (Fig. 6). Results of a single representative dot blot experiment representing one antibody to each of six gB structural domains are shown in Fig. S2.

10.1128/mSphere.00826-19.2 FIG S2 HSV-1 gC facilitates acid-induced conformational changes in the fusion protein gB. Extracellular preparations of HSV-1 gCR or ΔgC (∼10^7^ genome copies) were treated with pHs ranging from 7.3 to 5.0 and blotted directly to nitrocellulose. Blots were probed with each of the indicated gB MAbs, H126, H1838, SS144, and H1359, at neutral pH followed by horseradish peroxidase (HRP)-conjugated anti-mouse secondary antibody. The antibody name is shown at the left, and the gB domain to which each MAb is directed is indicated in parentheses. These represent individual examples of experiments whose results were quantitated and averaged together with multiple similar independent determinations. Summarized quantitative results are depicted in Fig. 6. Download FIG S2, TIF file, 1.2 MB. Copyright © 2020 Komala Sari et al. 2020 Komala Sari et al. This content is distributed under the terms of the Creative Commons Attribution 4.0 International license.

10.1128/mSphere.00826-19.4 FIG S4 Domain structure of HSV-1 gB and location of MAb epitopes. (A) gB ectodomain trimer representing a postfusion conformation. (B) Location of monoclonal antibody-binding sites. Monoclonal antibody-resistant mutations in domain I, which contains bipartite hydrophobic fusion loops, map to amino acid residue 303 for H126 and residues 203, 335, and 199 for SS55 (82, 83). The MAb H1781 epitope in domain II maps to residues 454 to 473, and H1838 maps to residues 391 to 410 (48). The H1359 epitope in domain III maps to residues 487 to 505 (74). SS10 in domain IV maps to residues 640 to 670 (48), and SS106 and SS144 in domain V both bind to residues 697 to 725 (54). The MAb H1817 epitope in domain VI (not resolved in the structure) maps to residues 31 to 43 (48). Download FIG S4, TIF file, 1.6 MB. Copyright © 2020 Komala Sari et al. 2020 Komala Sari et al. This content is distributed under the terms of the Creative Commons Attribution 4.0 International license.

As a reference for comparing HSV-1 ΔgC to HSV-1 gCR, the pH treatment that reduced MAb reactivity by >50% is indicated (Fig. 6). Domain I MAb H126 showed reduced reactivity with gB from HSV-1 gCR that had been subjected to acid treatment but, interestingly, exhibited little reduction with gB from HSV-1 ΔgC that had been similarly treated (Fig. 6). Using 50% reactivity as a reference point, in the absence of gC, the pH at which changes in the accessibility of the H126 gB epitope occurred was reduced by at least 0.6 pH units (Fig. 6). Similar results were obtained with SS55, another MAb to gB domain I. For SS55, the pH of gB conformational change in HSV-1 ΔgC was reduced by ∼0.5 pH units relative to gCR (Fig. 6). We have not previously examined the effect of low pH on gB domain II. Interestingly, MAbs to domain II, namely, H1781 and H1838, showed reduced binding to gCR virions that had been treated with mildly acidic pH, suggesting that gB domain II undergoes pH-triggered conformational change (Fig. 6; see also Fig. S2). The pH of antigenic change in both of the domain II epitopes tested was decreased in the absence of gC (Fig. 6). H1838 reactivity with gB in the ΔgC virus was particularly resistant to low pH; treatment with pH 5.0, the lowest pH tested, still resulted in >50% reactivity, suggesting that gC alters the pH of antigenic change in the H1838 epitope of gB by >0.7 pH units (Fig. 6). Domain III MAb H1359 had >50% reduced reactivity only with gB from HSV-1 gCR that had been treated under the most acidic condition tested, i.e., pH 5.0. The H1359 epitope in HSV-1 ΔgC was more resistant to pH 5.0 treatment than that in HSV-1 gCR.

Acidic pH triggers specific but not global changes in gB conformation, as low pH does not induce changes in the SS10 (domain IV) or H1817 (domain VI) epitopes of gB (16). The SS10 and H1817 epitopes in both gCR and ΔgC viruses were not altered by pH (Fig. 6). In contrast, gB domain V from wild-type HSV is known to undergo pH-induced conformational change. Following pH 5.0 treatment, domain V MAbs SS106 and SS144 showed a >50% reduction in reactivity with gB from both HSV-1 gCR and HSV-1 ΔgC, but gB from HSV-1 ΔgC was more resistant. This suggests that gC has an effect on low-pH-triggered change in gB domain V (Fig. 6).

A control deletion of a viral envelope glycoprotein gene other than the gC gene did not alter the pH of gB antigenic change of a representative epitope (Fig. S3). The gB H126 epitope (domain I) in HSV that lacks gE (HSV-1 F-gE/GFP [green fluorescent protein]) underwent pH-induced changes similar to those shown by gB from wild-type virus strain F. As a control, the H1817 (domain VI) epitope was unaffected by low pH regardless of the presence of gE (Fig. S3). Together, the results suggest that HSV-1 gC specifically increases the pH threshold of gB conformational change, particularly in domains I and II. This is consistent with gC facilitating low-pH entry and infectivity of HSV (Fig. 1, 2, and 5), possibly at the level of fusion with an endosomal membrane. Cargo transiting the host lysosome-terminal endocytosis pathway is subjected to decreasing pH (from ∼6.5 to 4.5). HSV colocalizes with endocytosis markers, but the specific fusion compartment involved has not been identified (8, 44). Intracellular transport of gC-negative HSV may be delayed because transit to a lower pH compartment is necessary for fusion-associated changes in gB.

10.1128/mSphere.00826-19.3 FIG S3 HSV-1 gE does not influence acid-induced conformational change in the H126 epitope of gB. (A) HSV-1 wild-type strain F or its gE-null (gE-GFP) derivative was treated with the indicated pHs and then directly blotted onto a nitrocellulose membrane. The blot was probed with representative gB MAb H126 or MAb H1817 at neutral pH. (B) Antibody reactivity was quantitated, and treatment with pH 7.4 was set as 100%. Data shown are representative of results from at least two independent experiments. Download FIG S3, TIF file, 0.6 MB. Copyright © 2020 Komala Sari et al. 2020 Komala Sari et al. This content is distributed under the terms of the Creative Commons Attribution 4.0 International license.

### The pH threshold of reversibility of conformational changes in gB.

**FIG 7:**
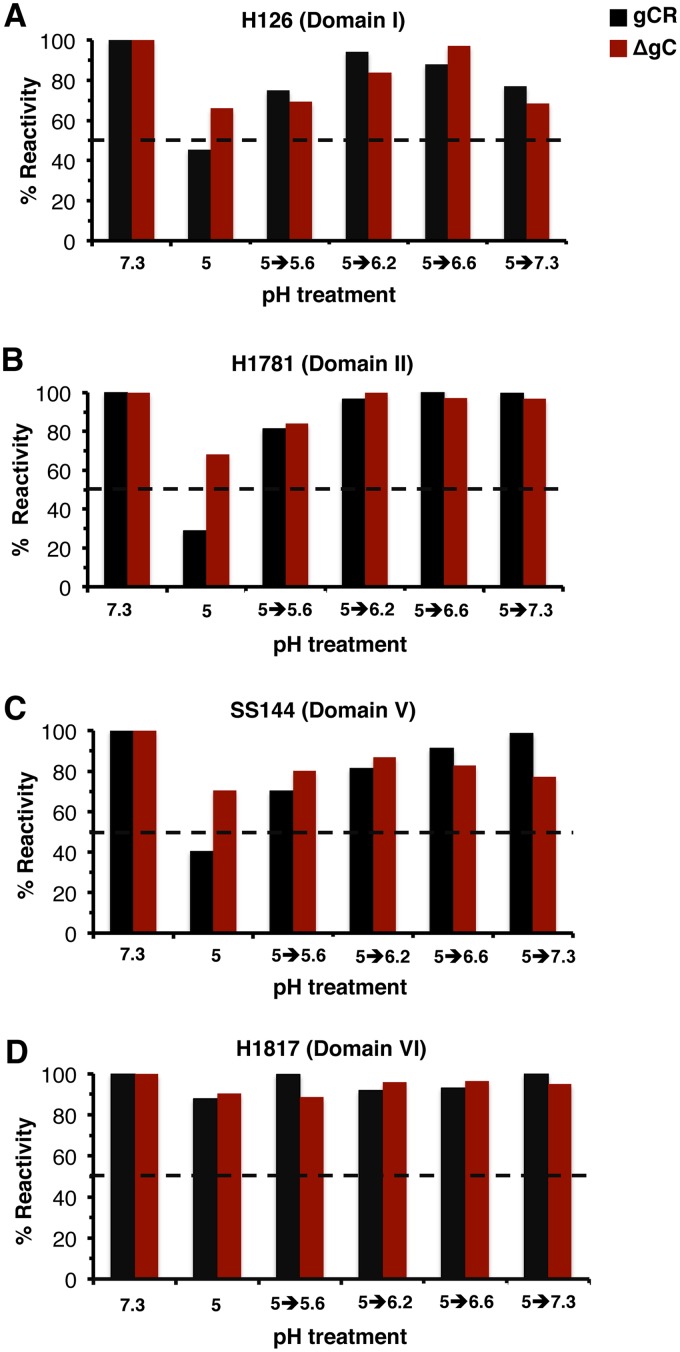
The pH threshold of reversibility of gB conformational changes. HSV-1 ΔgC or gCR was treated with pH 7.3 or 5.0 and incubated for 10 min. The pHs of the pH 5-treated samples were increased to the indicated levels for an additional 10 min. Samples were directly blotted to nitrocellulose and probed at neutral pH with gB MAb H126 (A), H1781 (B), SS144 (C), or H1817 (D). MAb reactivity was quantitated, with the pH 7.3-treated sample set to 100%. Data shown are representative of results from two independent experiments.

Reversibility of pH-triggered changes is a hallmark of gB and other class III fusion proteins (22, 45, 46). Prefusion and postfusion forms of class III proteins are proposed to exist in a pH equilibrium that is shifted to the postfusion state by acidic pH (47). During HSV egress, reversibility may allow gB on progeny virions to avoid nonproductive activation during transport through low-pH secretory vesicles. The pH threshold of initiation of gB conformational change is ∼6.2 to 6.4 (22). The pH at which acid-treated gB reverts to a pH-neutral conformation is not known. To determine this, virions were treated with pH 5 or maintained at pH 7.3 for 10 min at 37°C. The pH 5-treated samples were subjected to different target pHs for 10 min at 37°C to determine the pH at which reversibility occurs. Samples were then blotted to membrane and probed with representative MAbs to gB (Fig. 7). Upon treatment with a low pH of 5, there was reduced reactivity of MAb H126 (domain I) (Fig. 7A), H1781 (domain II) (Fig. 7B), and SS144 (domain V) (Fig. 7C) but not of MAb H1817 (domain VI) (Fig. 7D). Consistent with the data shown in Fig. 6, the reduction of gB antibody reactivity with HSV-1 ΔgC was less pronounced. Upon increasing the pH of gCR from 5.0 to 5.6, there was a partial restoration of gB antibody reactivity. Increasing the pH to 6.2 to 6.6 resulted in restoration of >80% of the reactivity measured at pH 7.3. This suggests that the threshold of reversibility of HSV gB conformational change is between pH 5.0 and 5.6. gB appears to be conformationally labile in this pH range.

To assess the influence of gC on the reversibility of gB conformational changes, ΔgC virions were tested for the reversibility of pH-triggered changes in the H126, H1781, SS144, and H1817 epitopes of gB. The results suggest that gC had little influence on the reversibility of gB conformational changes as measured here (Fig. 7A to C); rather, gC had a greater impact on the initial low-pH-induced antigenic changes in gB (Fig. 6; see also Fig. S2).

### Mildly acidic pH changes in gB oligomeric conformation are independent of gC.

**FIG 8:**
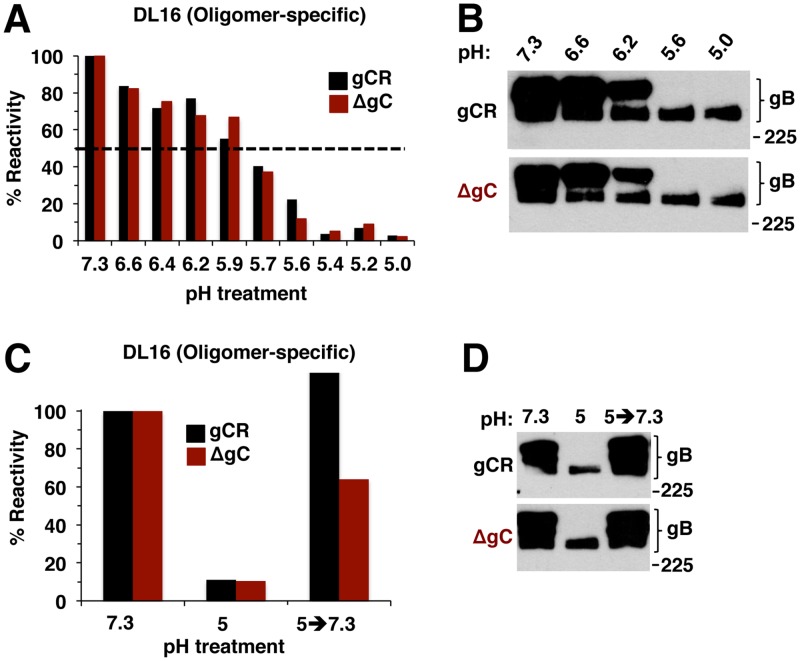
Low-pH-induced changes in gB oligomer are independent of gC, as is the reversibility of those changes. (A) HSV-1 gCR or ΔgC was treated with a range of pHs for 10 min. Samples were directly blotted to nitrocellulose and probed at neutral pH with gB oligomer-specific MAb DL16. Reactivity was quantitated, and the level seen with the pH 7.3 sample was set as 100%. (B) HSV-1 gCR or ΔgC was treated with a range of pHs for 10 min. SDS (1%) was added, and reactions were added to “native” PAGE sample buffer. Unheated samples were resolved by 8% SDS-PAGE and Western blot for HSV-1 gB. (C) HSV-1 gCR or ΔgC was treated with pH 5 for 10 min and then blotted directly to nitrocellulose or first neutralized back to pH 7.3 for 10 min and then blotted. Blots were probed at neutral pH with anti-gB MAb DL16. (D) HSV-1 gCR or ΔgC was treated with pH 5 for 10 min. One set of samples was neutralized back to pH 7.3. SDS (1%) was added, and samples were processed as described for panel B. Molecular size markers are indicated in kilodaltons at the right. Data shown are representative of results from at least two independent experiments.

An independent indicator of pH-induced alterations in gB is a shift to a lower-density gB oligomer in response to acid treatment (22). The role of gC in gB oligomeric changes was tested with MAb DL16, which recognizes an oligomer-specific epitope within gB domain V (48). When either HSV-1 ΔgC or gCR was pretreated with low pH, the levels of DL16 reactivity were similarly reduced (Fig. 8A), distinguishing the DL16 epitope on gB from the other acid-sensitive epitopes (Fig. 6). This result signifies acid-triggered change in the oligomeric conformation of gB (Fig. 8A) regardless of the presence or absence of gC. This outcome was confirmed by an independent measure of gB oligomeric conformation. When HSV is first treated with low pH and then subjected to 1% SDS and native PAGE, the slower-migrating, higher-molecular-weight (HMW) species of gB oligomer disappears, suggesting a change in gB oligomeric conformation (22). Using this approach, the HMW gB oligomer from HSV-1 ΔgC disappeared in a manner similar to that seen with gB from the control rescuant virus gCR (Fig. 8B). Although gC regulates low-pH induced antigenic changes in gB (Fig. 6), it does not appear to affect acid-triggered alterations in the gB oligomer. This is consistent with the notion that similarly low pH levels trigger both antigenic and oligomeric alterations in gB but that these changes are experimentally separable and not identical (24).

Since the changes in gB oligomeric conformation did not require gC, we expected that their reversibility would also be independent of gC. This was indeed the case. Acid-induced reduction of reactivity with the oligomer-specific MAb DL16 was partially reversible in both ΔgC and gCR viruses (Fig. 8C). Likewise, the susceptibility of low-pH-treated gB oligomer to disruption by 1% SDS was also reversible, regardless of the presence or absence of gC (Fig. 8D). Thus, the reversibility of low-pH-triggered conformational changes in the gB oligomer is independent of gC.

## DISCUSSION

Knowledge of the mechanisms underlying how herpesviruses traverse distinct cellular entry pathways is paramount for our understanding of these important pathogens. The experiments described here reveal a selective role for HSV-1 gC in low-pH entry. HSV-1 gC confers an infectivity advantage in cells that support low-pH entry of HSV-1, such as human keratinocytes. There is a lag in the exit of enveloped gC-negative particles from endocytic compartments, which may reflect a role for gC in optimal virus trafficking or low-pH fusion. Low-pH-triggered antigenic changes in gB domains I, II, and V are thought to be critical for fusion (22, 40, 41, 49–51) (Fig. 6; see also Fig. S2 in the supplemental material). gC facilitates pH-induced gB conformational changes, increasing the pH of antigenic change by as much as 0.4 to 0.7 pH units. The reduced entry of gC-negative HSV may be explained by the role of gC in facilitating fusion-associated conformational changes in gB, which result in optimal penetration from an endocytic compartment. Importantly, in the absence of gC, HSV still uses a low-pH pathway to enter and infect cells, likely mediated in part by changes in gB that occur in the absence of gC, albeit at a lower pH.

We very recently demonstrated that HSV-1 gC protects gB from neutralizing antibodies (38). HSV-1 gC also binds to complement component C3b and inhibits complement-mediated immunity (52). When HSV particles were treated with soluble heparin in a cell-free assay, the UL16 tegument protein was rearranged in a gC-dependent manner (53). The role of gC in entry mediated by an acidic endosomal pathway as described here is independent of its role in attachment to cell surface heparan sulfate.

Viral envelope glycoprotein B (gB) is highly conserved among all subfamilies of the *Herpesviridae*. Current models of HSV-1 entry posit that (i) gC (and, to a lesser extent, gB) mediates viral attachment to host cell surface glycosaminoglycans and that (ii) gD binds to a cognate host cell receptor such as HVEM or nectin-1, resulting in pH-independent conformational change in gD; this is thought to (iii) transmit a signal to gH/gL and (iv) culminate in the execution of membrane fusion by gB. Thus, gB, unlike other members of the class III fusion protein family, does not mediate fusion on its own. In addition to pH-triggered changes in gB domains I and V, we show here that acid-induced antigenic changes also occur in domain II (Fig. 6; see also Fig. S2).

There is structure-based evidence that gB can exist in multiple conformations. The postfusion structure of gB is known, and distinct, membrane-associated nonpostfusion forms that may reflect the prefusion conformation have also been resolved (54–56). MAbs that bind specifically to either prefusion or postfusion gB have not been identified. Antibody binding to prefusion gB present in virions that are pretreated with low pH does not disappear completely; instead, there are decreases in antibody reactivity. Low pH causes gB to assume a nonprefusion form but is likely not sufficient to shift gB to the postfusion form. In the absence of gC, the pH threshold for gB conformational changes, particularly in domain I and II, is lower by 0.4 to 0.7 pH units (Fig. 6). In comparison, variants and mutants of influenza virus hemagglutinin (HA) exhibit an approximately 0.2 to 0.6 shift in the pH associated with both conformational change and fusion (57, 58). The H126 epitope in the fusion domain of gB might be particularly important for the pH activation of fusion (24). In the absence of gC, a more acidic pH is required to trigger changes in the accessibility of the H126 epitope.

The cell tropism of herpesvirus entry and infection is influenced by subfamily-specific viral proteins. Epstein-Barr virus (EBV) gp42 is required for fusion and entry in B cells but not epithelial cells (59–63). The human cytomegalovirus (HCMV) pentamer complex of envelope proteins is necessary for endosomal entry into epithelial and endothelial cells but not for pH-neutral entry into fibroblasts (10, 11, 64–66). However, the details of the mechanism underlying the role of EBV gp42 or the HCMV pentamer in selection of the entry pathway are not known. Alphaherpesvirus-specific protein gC is the first HSV envelope protein to have been reported to selectively participate in endocytic entry. Here, we suggest that gC is important for HSV-1 epithelial infection but is less so for neuronal entry. The results are consistent with a mechanism whereby gC acts to ensure that gB undergoes optimal conformational change to mediate fusion with an appropriate endosomal compartment.

The results suggest a functional interaction between gC and gB. Direct interaction between gC and gB has not been detected by coimmunoprecipitation approaches at different pHs (data not shown). Low-affinity or transient interactions may not be captured, or gC may exert an indirect effect on gB through another viral or host factor. Physical interactions between HSV-1 gB and gH have also been difficult to detect, despite previous demonstrations of functional interactions (20, 21, 67). Future investigation of gC-gB interactions could include split-fluorescent protein, fluorescence resonance energy transfer (FRET), or proximity ligation approaches. We propose a model whereby gC aids gB and, together with gD and gH/gL, allows rapid entry of HSV-1 into epithelial cells.

## MATERIALS AND METHODS

### Cells and viruses.

Human HaCaT epithelial keratinocytes and Vero cells were propagated in Dulbecco’s modified Eagle’s medium (DMEM; Thermo Fisher Scientific) supplemented with 10% fetal bovine serum (FBS; Atlanta Biologicals). Nondifferentiated human SK-N-SH and IMR-32 neuroblastoma cells (ATCC) were propagated in Eagle’s minimal essential medium supplemented with 10% FBS, 1 mM sodium pyruvate, 0.1 mM nonessential amino acids, and Earle’s salts (Invitrogen). CHO-HVEM (M1A) cells (68) (provided by R. Eisenberg and G. Cohen, University of Pennsylvania) are stably transformed with the human HVEM gene and contain the Escherichia coli *lacZ* gene under the control of the HSV-1 ICP4 gene promoter. CHO-HVEM cells were propagated in Ham’s F-12 nutrient mixture (Gibco/Life Technologies) supplemented with 10% FBS, 150 μg of puromycin (Sigma-Aldrich, St. Louis, MO, USA)/ml, and 250 μg of G418 sulfate (Thermo Fisher Scientific, Fair Lawn, NJ, USA)/ml. Cells were subcultured in nonselective medium prior to use in all experiments. Primary human epidermal keratinocytes (HEKa) (ATCC) were maintained up to passage 8 in dermal cell basal medium (ATCC) supplemented with a keratinocyte growth kit (ATCC) and penicillin-streptomycin-amphotericin B solution (ATCC). CHOpgs745 cells (ATCC), which lack a gene required for heparan sulfate biosynthesis, were propagated in Ham’s F-12 nutrient mixture supplemented with 10% FBS.

HSV-1 strain KOS and all viruses in this study were propagated and titers were determined on Vero cells. HSV-1 genome copy numbers were determined by qPCR (69). HSV-1 (KOS) ΔgC2-3 or HSV-1 ΔgC is HSV-1 KOS in which most of the gC gene was deleted and replaced by *lacZ* (33); this virus is considered a gC-negative HSV-1 strain. HSV-1 (KOS) gC2-3Rev virus or HSV-1 gCR is a recombinant in which HSV-1 (KOS) ΔgC2-3 was rescued by insertion of the wild-type gC gene (33). Both viruses were obtained from C. Brandt, University of Wisconsin—Madison. The viral genomes have not been sequenced. However, HSV-1 gCR and the parental KOS strain are indistinguishable in terms of specific infectivity, cell attachment, heparan sulfate binding, and infectivity. Their genomes are similar as measured by restriction enzyme analysis and Southern blotting (33). In addition, the phenotypes attributed to HSV-1 gCR in this study are similar to those previously reported for HSV-1 wild-type strain KOS (6–8, 22, 24, 70, 71). HSV-1 F-gE/GFP (obtained from D. Johnson, Oregon Health Sciences University) lacks the gE gene (72).

### Antibodies.

Anti-gB mouse monoclonal antibodies H126 (domain I), H1359 (domain III), and H1817 (domain VI) were from Virusys. Anti-gB monoclonal antibodies DL16 (oligomer specific; domain V), SS10 (domain IV), SS55 (domain I) (73), SS106 (domain V), and SS144 (domain V) (48) were provided by G. Cohen and R. Eisenberg, University of Pennsylvania. Anti-gB monoclonal antibodies H1838 and H1781 (domain II) were provided by L. Pereira, University of California, San Francisco (74).

### Immunofluorescence assay of HSV entry and infectivity.

Equivalent amounts of HSV-1 ΔgC or gCR were added to cells grown on coverslips in 24-well plates in triplicate. Cells were incubated at 4°C for 1 h and then washed twice with cold phosphate-buffered saline (PBS). Cultures were then shifted to 37°C in normal culture medium for 6 h and then fixed with 100% ice-cold methanol. Primary antibody H1A021 to HSV-1 ICP4 (Virusys) was then added, followed by Alexa Fluor-488-labeled secondary antibody. Nuclei were counterstained with 12.5 ng/ml 4′,6-diamidino-2-phenylindole (DAPI). Approximately 500 cells per well were counted and scored for successful infection.

### HSV-1 plating efficiency.

Ten-fold dilutions of HSV-1 stock were added to SK-N-SH or HaCaT cells at 37°C. At 18 to 20 h p.i., cells were fixed with ice-cold methanol and acetone (2:1 ratio) for 20 min at −20°C and air-dried. Titers were determined by a limiting dilution, immunoperoxidase plaque assay with rabbit polyclonal antibody to HSV, HR50 (Fitzgerald Industries, Concord, MA). Following three washes with 0.5% Tween 20–PBS, a 1:200 dilution of goat anti-rabbit IgG conjugated with horseradish peroxidase (Thermo Fisher Scientific) was added for 2 h at room temperature. Following three washes with 0.5% Tween 20–PBS, 4-chloro-1-naphtol (Sigma) substrate was added. Plaques were visualized with a Leica stereoscope, and titers were calculated.

### Ectopic expression of HSV-1 gC.

Lipofectamine 3000 (Thermo Fisher Scientific) in serum-free Opti-MEM (Thermo Fisher Scientific) was used to transfect cells with plasmids encoding gC (pSH140; obtained from G. Cohen and R. Eisenberg [75]) or gD (pPEP99; obtained from P. Spear [76]) or with empty vector for 48 h at 37°C. Transfected cells were infected with 10-fold dilutions of HSV-1. At 18 h p.i., titers were determined by plaque assay.

### SDS-PAGE and Western blotting.

HSV-1 was boiled in Laemmli buffer containing 200 mM dithiothreitol for 5 min. Proteins were separated by SDS-PAGE on Tris-glycine gels (Thermo Fisher Scientific). For protein staining, gels were then fixed and stained with 0.025% Coomassie brilliant blue (J. T. Baker Chemical Co., Philipsburg, NJ), 40% methanol (Baker Chemical), and 10% glacial acetic acid (Baker Chemical), followed by destaining with 30% methanol and 7% glacial acetic acid (71). Gels were dried and imaged with a Gel Doc XR imager (Bio-Rad, Hercules, CA). For Western blotting, following transfer to nitrocellulose, membranes were blocked and incubated with HSV polyclonal antibodies R68 (anti-gB), R47 (anti-gC) (77), R2 (anti-gD) (78), R137 (anti-gH), or anti-gD monoclonal antibody DL6 (79), which were gifts from G. Cohen and R. Eisenberg; with HSV-1 gE monoclonal antibody H1A054-100 (Virusys); or with monoclonal antibody H1A021 to VP5 (Santa Cruz Biotechnology, Dallas, TX). After incubation with horseradish peroxidase-conjugated secondary antibodies, enhanced chemiluminescent substrate (Pierce) was added, and membranes were exposed to X-ray film (Kodak).

### Effect of ammonium chloride on HSV entry and infectivity.

Cells grown in 24-well plates were treated with medium containing ammonium chloride for 1 h at 37°C. Virus was added in the continued presence of the agent for 6 h. The medium was then removed and replaced with complete DMEM. At 16 h p.i., a plaque assay was performed to measure infectivity. CHO-HVEM cells grown in 96-well plates were treated with medium containing ammonium chloride for 20 min at 37°C. Virus was added (multiplicity of infection [MOI] of 5) in the continued presence of the agent, and the beta-galactosidase activity of cell lysates was measured at 6 h p.i.

### Attachment of HSV-1 to the cell surface.

Cells grown in 96-well plates were prechilled in carbonate-free, serum-free medium supplemented with 20 mM HEPES and 0.2% bovine serum albumin (binding medium) at 4°C on ice for 20 min. Extracellular preparations of approximately 10^6^ genome copies of HSV-1 ΔgC or gCR in ice-cold binding medium were added to 3.4 × 10^4^ to 7 × 10^4^ cells at 4°C on ice for 1 h. Cultures were washed twice with ice-cold PBS. Cells were trypsinized, and cell-associated HSV-1 DNA was isolated with a QIAamp DNA blood minikit (Qiagen) according to the manufacturer’s instructions. Attached (cell-associated) HSV-1 was quantitated by qPCR as previously described (17, 69).

### Intracellular tracking of enveloped, infectious HSV.

HSV-1 ΔgC or gCR was added to confluent cell monolayers (MOI of 8) on ice at 4°C for 2 h. Cultures were washed with PBS and shifted to 37°C. At the indicated times p.i., extracellular virus was inactivated by adding sodium citrate buffer (pH 3.0) for 1 min at 37°C (6). Monolayers were immediately put on ice and washed with cold PBS. One milliliter of Ham’s F12 medium mixed with 20 mM HEPES and 1% FBS was added, and cells were lysed by two cycles of freezing and thawing. Titers of lysates were determined on Vero cells.

### Dot blot analysis of HSV-1 gB.

Extracellular preparations of HSV-1 were diluted in serum-free, bicarbonate-free DMEM with 0.2% bovine serum albumin (BSA) and 5 mM (each) HEPES, MES (morpholineethanesulfonic acid), and sodium succinate. Virions were adjusted with HCl to achieve pHs ranging from 5.0 to 7.3. Approximately 10^7^ genome copies of HSV-1 were incubated at 37°C for 10 min and were then either blotted directly to a nitrocellulose membrane using a Minifold dot blot system (Whatman) or were first neutralized by addition of pretitrated amounts of 0.05 N NaOH (80). Virus-dotted nitrocellulose membranes were blocked and then incubated with antibodies to gB at neutral pH. After incubation with horseradish peroxidase-conjugated secondary antibodies, enhanced chemiluminescent substrate (Thermo Fisher Scientific) was added, and blots were exposed to X-ray film (Genesee Scientific). Densitometry was performed with ImageJ.

### Analysis of gB oligomeric structure by PAGE.

Extracellular preparations of HSV were diluted in the same medium as was used for dot blot analysis. Virus samples were adjusted to the indicated pHs with pretitrated amounts of 0.05 N HCl for 10 min at 37°C. To test for reversibility of conformational change, samples were then neutralized by addition of pretitrated amounts of 0.05 N NaOH for 10 min at 37°C. SDS (1%) was then added. Laemmli sample buffer containing 0.2% sodium dodecyl sulfate (SDS) with no reducing agent (81) was added. Samples were not heated, and proteins were resolved by PAGE. After transfer to nitrocellulose, membranes were blocked and incubated with gB monoclonal antibody H1359. After incubation with horseradish peroxidase-conjugated secondary antibody, enhanced chemiluminescent substrate (Thermo Fisher) was added and membranes were exposed to X-ray film (Genesee Scientific).
