# Supplementary figures and images for: Herpes Simplex Virus Glycoprotein C Regulates Low-pH Entry
Source: mSphere. 2020 Feb 5;5(1):e00826-19. doi: 10.1128/mSphere.00826-19 (PMC7002311; doi:10.1128/mSphere.00826-19)

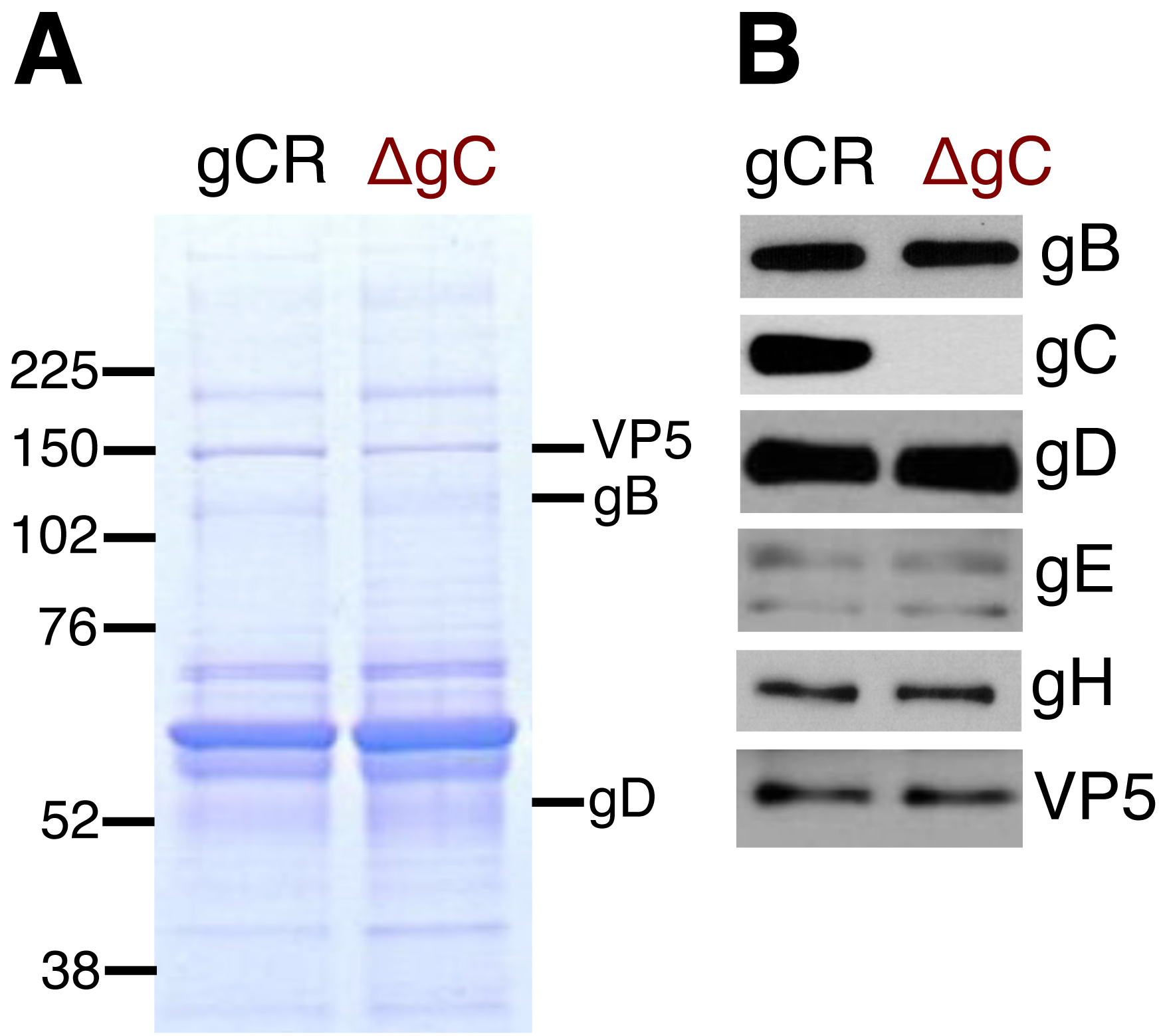

Supplement: FIG S1 [file mSphere.00826-19-sf001.tif]

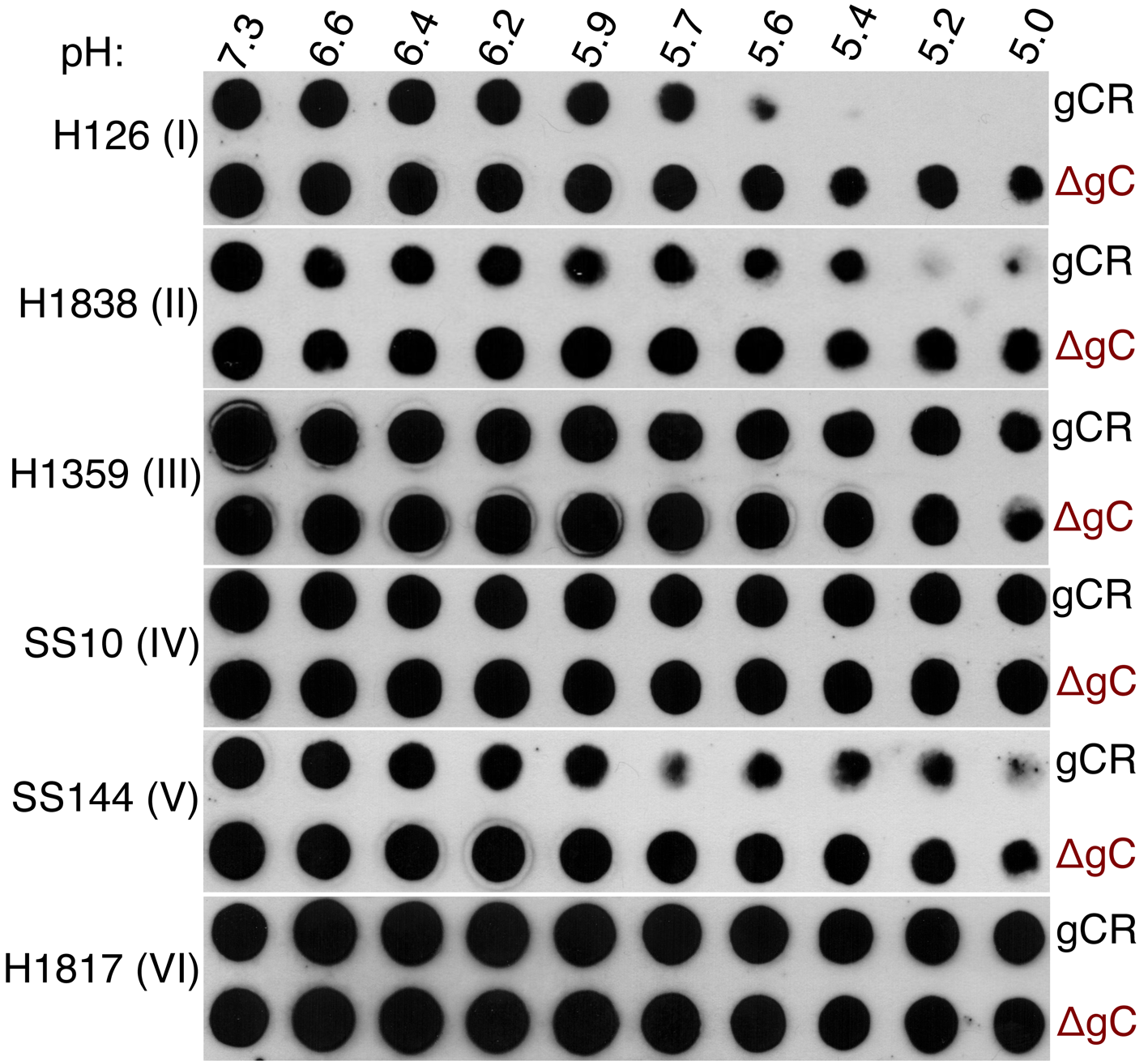

Supplement: FIG S2 [file mSphere.00826-19-sf002.tif]

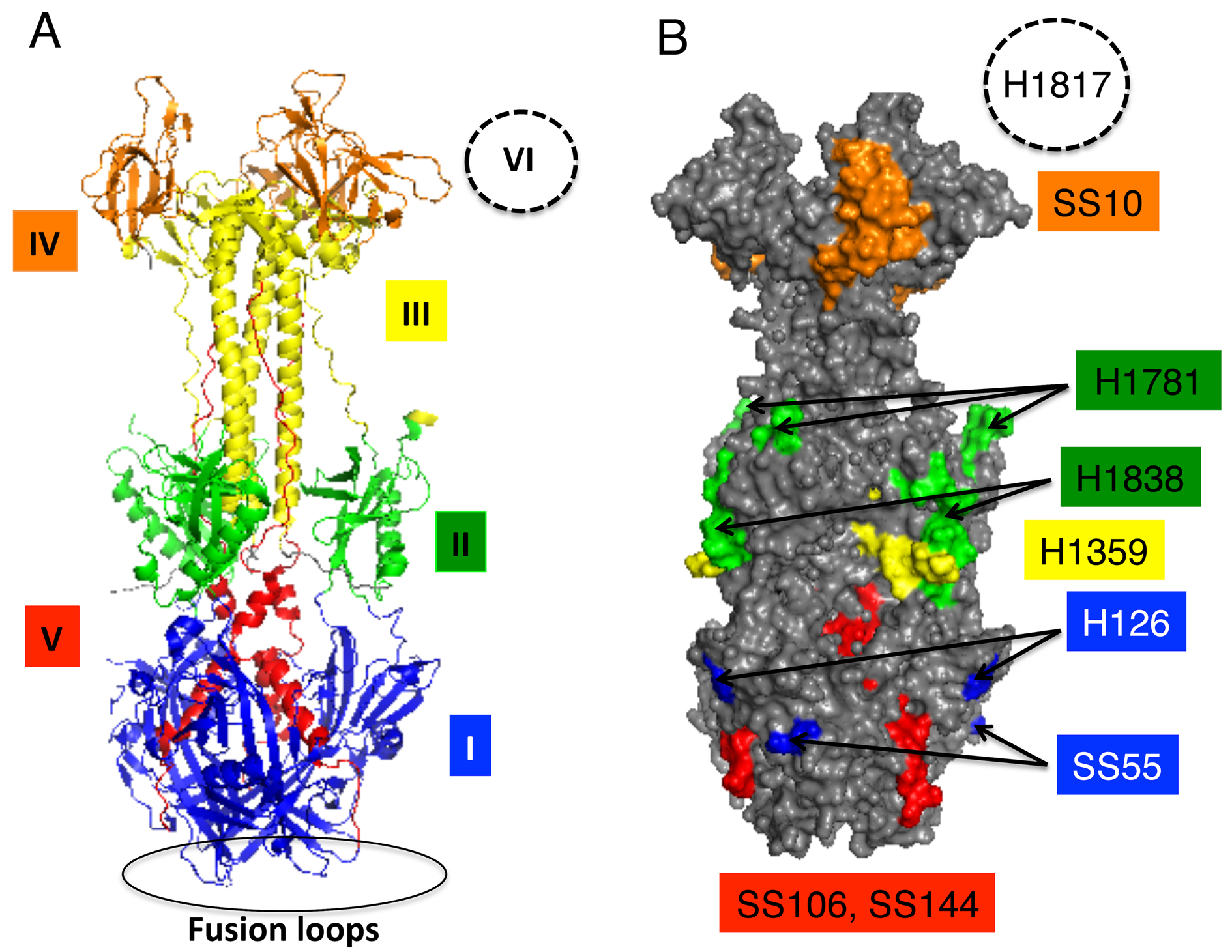

Supplement: FIG S4 [file mSphere.00826-19-sf004.tif]

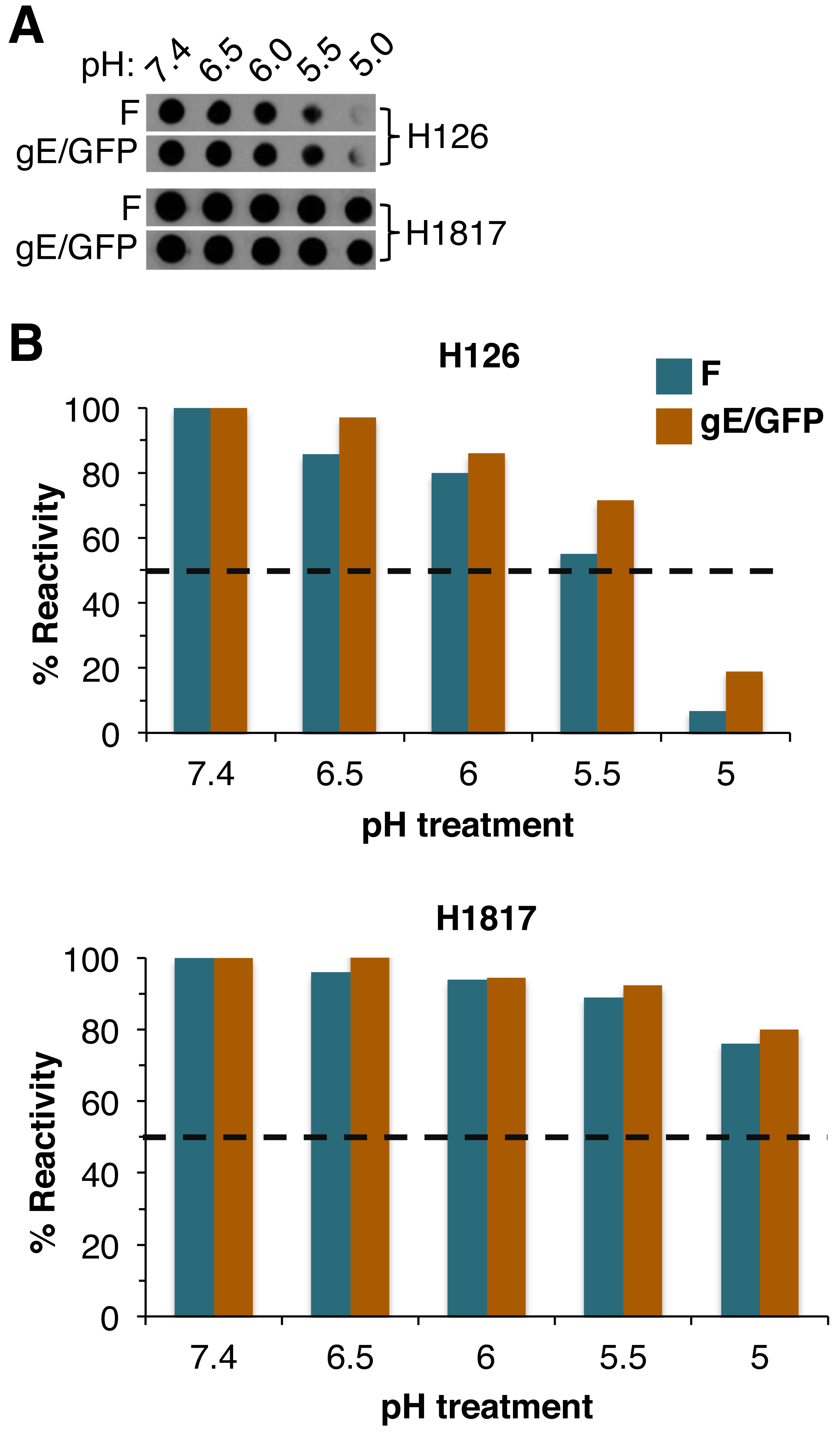

Supplement: FIG S3 [file mSphere.00826-19-sf003.tif]
